# Delays in Tuberculosis Treatment Among People With Pulmonary Tuberculosis in East Africa: Findings From a Prospective Cohort Study

**DOI:** 10.1093/ofid/ofag392

**Published:** 2026-07-10

**Authors:** Kirsten Prabhudas-Strycker, Lameck Diero, Winnie Muyindike, Helen Byakwaga, Sylvia Kitur, Joseph Mining’wo, Bob Ssekyanzi, Alexis Byaruhanga, Harold Kooreman, Felix Pabon-Rodriguez, Hong-Ha M Truong, Jayne Lewis Kulzer, Edwin Ochomo, Francesca Odhiambo, Suzanne Goodrich, Aggrey Semeere, Neelima Navuluri, Kara Wools-Kaloustian, Leslie A Enane

**Affiliations:** The Ryan White Center for Pediatric Infectious Disease and Global Health, Department of Pediatrics, Indiana University School of Medicine, Indianapolis, Indiana, USA; Division of Infectious Diseases, Department of Medicine, Indiana University School of Medicine, Indianapolis, Indiana, USA; Department of Medicine, Moi University College of Health Sciences, Eldoret, Kenya; Academic Model Providing Access to Healthcare (AMPATH), Eldoret, Kenya; Department of Internal Medicine, Faculty of Medicine, Mbarara University of Science and Technology, Mbarara, Uganda; Mbarara Regional Referral Hospital, Mbarara, Uganda; Infectious Diseases Institute, Makerere University College of Health Sciences, Kampala, Uganda; Academic Model Providing Access to Healthcare (AMPATH), Eldoret, Kenya; Academic Model Providing Access to Healthcare (AMPATH), Eldoret, Kenya; Mbarara Regional Referral Hospital, Mbarara, Uganda; Mbarara Regional Referral Hospital, Mbarara, Uganda; Department of Biostatistics and Health Data Science, Indiana University School of Medicine, Indianapolis, Indiana, USA; Department of Biostatistics and Health Data Science, Indiana University School of Medicine, Indianapolis, Indiana, USA; Department of Medicine, University of California, SanFrancisco, San Francisco, California, USA; Department of Obstetrics, Gynecology and Reproductive Sciences, University of California, SanFrancisco, San Francisco, California, USA; Centre for Microbiology Research, Research Care and Training Program, Kenya Medical Research Institute, Nairobi, Kenya; Centre for Microbiology Research, Research Care and Training Program, Kenya Medical Research Institute, Nairobi, Kenya; Division of Infectious Diseases, Department of Medicine, Indiana University School of Medicine, Indianapolis, Indiana, USA; Academic Model Providing Access to Healthcare (AMPATH), Eldoret, Kenya; Infectious Diseases Institute, Makerere University College of Health Sciences, Kampala, Uganda; Division of Pulmonary, Allergy, and Critical Care Medicine, Department of Medicine, Duke University School of Medicine, Durham, North Carolina, USA; Duke Global Health Institute, Duke University, Durham, North Carolina, USA; Division of Infectious Diseases, Department of Medicine, Indiana University School of Medicine, Indianapolis, Indiana, USA; Academic Model Providing Access to Healthcare (AMPATH), Eldoret, Kenya; Center for Global Health, Indiana University, Indianapolis, Indiana, USA; The Ryan White Center for Pediatric Infectious Disease and Global Health, Department of Pediatrics, Indiana University School of Medicine, Indianapolis, Indiana, USA; Center for Global Health, Indiana University, Indianapolis, Indiana, USA

**Keywords:** case-finding, diagnostic delays, TB care cascade, HIV, Tuberculosis

## Abstract

**Background:**

Although timely tuberculosis (TB) diagnosis and treatment are essential to TB elimination, care delays remain a challenge. Understanding factors associated with delayed TB diagnosis and treatment may inform interventions.

**Methods:**

The TB Sentinel Research Network of the International epidemiology Databases to Evaluate AIDS (IeDEA) is a prospective study of people aged ≥15 years with pulmonary TB. At sites in Eldoret, Kenya, and Mbarara, Uganda, questionnaires ascertained timing of recalled TB symptom onset, number/setting of healthcare visits prior to TB diagnosis, symptoms, demographics, food insecurity, and TB- and HIV-related stigma. Robust negative binomial regression was used to examine factors associated with longer overall duration from TB symptom onset to documented treatment initiation.

**Results:**

Among 264 participants—median age 33 years (IQR, 25–43 years), 67% male, 31% people with HIV, 51% urban residents—median duration from TB symptom onset to treatment initiation was 65.5 days (IQR, 33–131 days). Longer time to TB treatment was associated with female sex (adjusted incidence rate ratio [aIRR], 1.27 [95% CI, 1.00–1.61]); previous care-seeking at a pharmacy (aIRR, 1.53 [95% CI, 1.22–1.92]), and presence of dyspnea (aIRR, 1.43 [95% CI, 1.09–1.86]) or fatigue (aIRR, 1.63 [95% CI, 1.11–2.37]) at initiation. Shorter duration to TB treatment was associated with HIV-positive status (aIRR, 0.50 [95% CI, .39–.64]). Among people with HIV, shorter duration to TB treatment was associated with CD4 count <200 cells/μL (aIRR, 0.63 [95% CI, .43–.93]).

**Conclusions:**

Interventions are needed to increase community-level TB diagnostic access, including at pharmacies and lower levels of care. Decentralized TB testing strategies and tailored interventions for groups with lower care access should be pursued.

## Teaser

In a prospective cohort of people aged ≥15 with pulmonary TB in East Africa, we found prolonged duration from symptom onset to treatment initiation. Women, people without HIV, and people seeking care at pharmacies were particularly affected by delayed care.

In 2024, an estimated 10.7 million people fell ill with tuberculosis (TB) and 1.23 million people died from TB [1]. Since 2015, TB incidence has decreased by only 12.3% and mortality by 29% [1]—well below the World Health Organization (WHO) End TB Strategy 2025 milestones of 50% and 75%, respectively [2]. Failure to meet these global targets resulted from years of incremental progress followed by COVID-19 pandemic–related reversals of previous trends. Pandemic-related disruptions to TB diagnosis and treatment resulted in increased TB incidence and an estimated 700 000 excess deaths from TB in 2020–2023 [1].

Delays in TB diagnosis and treatment initiation are a longstanding challenge in TB-endemic settings [3–7]. Longer duration from symptom onset to treatment initiation is associated with greater TB transmission [8, 9] poor treatment outcomes, and posttreatment morbidity [10, 11], including post-TB lung disease [12]. In East Africa, the rollout of WHO-recommended Xpert MTB/RIF for initial TB diagnosis resulted in increased bacteriological confirmation of TB and reduced diagnostic turnaround times [13, 14]. However, limitations to availability of Xpert testing at lower levels of care, financing challenges, stockouts, and other health system challenges have resulted in underutilization of Xpert [15]. Further socioeconomic and cultural barriers to TB testing access have been qualitatively described [16].

Understanding the ongoing drivers of delayed TB diagnosis and treatment initiation in East Africa is critical for ending TB. There is a need to investigate timing to TB care initiation and factors associated with delay in the current era since Xpert implementation and after the COVID pandemic. Incorporation of sociodemographic and behavioral measures, such as food insecurity and stigma, can inform present gaps in TB care access and needed interventions [5]. We therefore investigated duration from recalled TB symptom onset to treatment initiation, as well as demographic, sociodemographic, clinical, and behavioral factors associated with delayed TB care, in a prospective cohort of people with pulmonary TB in East Africa.

## METHODS

### Study Setting and Population

The TB Sentinel Research Network (TB-SRN) of the International epidemiology Databases to Evaluate AIDS (IeDEA) is a global prospective study of people aged ≥15 years with pulmonary TB, with or without human immunodeficiency virus (HIV) infection [17]. Participants are enrolled at TB treatment initiation, with detailed data collection at scheduled study visits during and after treatment.

East Africa TB-SRN sites are located at Mbarara Regional Referral Hospital (MRRH) in Mbarara Uganda, and at Moi Teaching and Referral Hospital (MTRH) in Eldoret, Kenya. HIV and TB services are co-located and integrated on the campuses of both of these major referral hospitals. MRRH serves a predominantly rural population in western Uganda. MTRH serves urban and rural populations in western Kenya and is the anchor institution of the Academic Model Providing Access to Healthcare (AMPATH) partnership [18]. At both sites, services for TB diagnosis and management are provided by the respective national TB programs, following WHO guidelines [13]. Sputum Xpert MTB/RIF is used as the initial diagnostic for pulmonary TB, and results are available the day after sputum collection. Patients providing samples are typically asked to return the next day for their results, and if positive, are started on treatment the same day. Clinically diagnosed TB (without biological confirmation) is also managed with same-day treatment initiation.

### Data Collection

Data for this study were collected from 21 February 2023 to 17 July 2025, via participant questionnaires and abstraction from medical records. Baseline data collection at time of TB treatment initiation included age, sex, HIV status, place of residence, educational level, and marital status. Abstracted clinical data included type of TB (pulmonary alone or pulmonary and extrapulmonary), microbiologic status, and hospitalization at TB presentation. For people with HIV (PWH), antiretroviral treatment (ART) status, viral load, and CD4 count were assessed. Self-reported dates of symptom onset and of first healthcare presentation were collected. If exact dates could not be recalled, participants were asked to provide a best approximation. Site of initial care presentation was collected, including, for example, pharmacy, private practice, private hospital, public district/provincial hospital, or tertiary/referral hospital. Standardized questionnaires asked about presence of TB symptoms (fever, cough, hemoptysis, night sweats, weight loss, chest pain, dyspnea, fatigue, loss of appetite, and abdominal pain) within the past 4 weeks. Questionnaires also ascertained history of other medical conditions and treatments, including known lung disease, cancer, and treatment with immunosuppressive therapy.

Data on food insecurity and TB and HIV stigma were collected at 1 month into TB treatment. Food insecurity was ascertained by the Household Food Insecurity Access Scale questionnaire. TB and HIV stigma were ascertained by adapted Van Rie scales for TB, perceived community HIV, and felt HIV stigma [19, 20].

### Analysis

**Figure 1:**
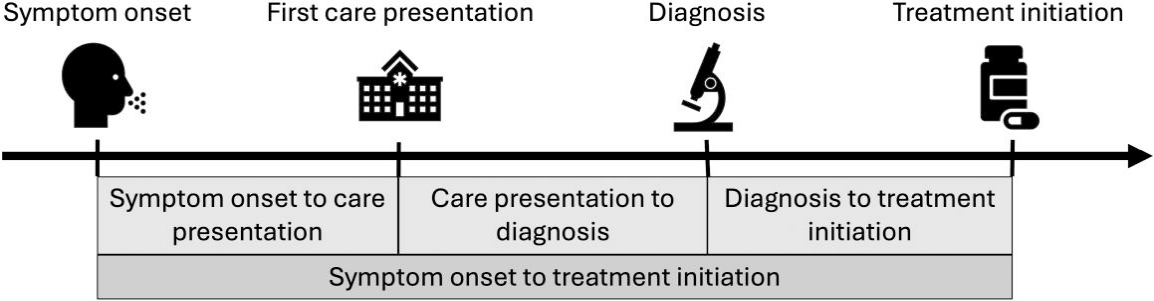
Time durations of interest for key events in the tuberculosis (TB) diagnosis and treatment initiation cascade described in this study. Time durations are graphically represented for the primary outcome of interest—duration from TB symptom onset to TB treatment initiation—and for secondary outcomes for the durations between key steps in this cascade.

We summarized participant characteristics using descriptive statistics. Time durations (in days) were described for key events in the TB diagnosis and treatment initiation cascade: from self-reported TB symptom onset to first healthcare presentation; from first healthcare presentation to TB diagnosis; and from TB diagnosis to TB treatment initiation (Figure 1). Overall time from symptom onset to treatment initiation was selected at the outset as the primary outcome for multivariable analyses given its clinical relevance and implication for transmission. Due to the positively skewed distribution and overdispersion of the primary outcome, we used robust negative binomial regression [21] to estimate incidence rate ratios (IRRs) for factors associated with longer duration to TB treatment initiation. We did not define a cut-off for delayed treatment initiation, keeping duration to TB treatment initiation as a continuous variable to incorporate the full information contained in the continuous variable [22]. Covariates included sociodemographic (eg, age, sex, urban/rural residence, education), clinical (eg, HIV status, TB symptoms, prior TB), and behavioral variables (eg, sites of prior healthcare-seeking, TB/HIV stigma measures). Predictors were selected based on clinical relevance and bivariate associations. Distance between participants’ residences and TB clinic was considered for inclusion in the model but was not associated with the outcome in bivariable analysis. The point-biserial correlation between rural/urban residence and distance to TB clinic was moderate to high, and we opted for inclusion of rural/urban residence over distance to TB clinic as distance to TB clinic may be influenced by other factors (eg, stigma leading to participants opting for more distant care to avoid recognition). Separate models were evaluated among the subset of PWH to explore the role of HIV-related stigma and clinical symptoms within this group. We used empirical cumulative distribution functions to visualize timing to TB treatment initiation overall and stratified by HIV status, sex, and age group. Sensitivity analyses included checks for multicollinearity and influential observations using Cook's distance and leverage diagnostics. Missing data were minimal, and were thus imputed via multivariate imputation by chained equations [23], and participants missing symptom onset or treatment initiation dates (n = 6/264) were excluded from regression analyses. All analyses were conducted using R version 4.4.3 software [24], with a significance level set at 5%.

### Ethics

This study was reviewed and approved by the MTRH/Moi University Institutional Research and Ethics Committee, the Mbarara University of Science and Technology Research Ethics Committee, and by the Indiana University Institutional Review Board, as well as by national regulatory authorities in Kenya and Uganda. All participants provided informed consent (and assent, for minors) to participate.

## RESULTS

### Sociodemographic and Clinical Characteristics

This analysis included 264 participants: 171 from Eldoret, Kenya, and 93 from Mbarara, Uganda (Table 1). Median age was 33 years (interquartile range [IQR], 25–43 years), 176 (67%) were male, 81 (31%) were PWH, and 134 (51%) were urban residents. For PWH, 67% were on ART at the time of TB diagnosis. Median CD4 cell count was 121 cells/μL, and HIV viral load suppression (<75 copies/mL) was documented among 19%. Among all participants, 235 (89%) had microbiologic confirmation of TB, 57 (22%) had previous history of TB disease, and 55 (21%) had a known contact with TB. Three participants had both extrapulmonary and pulmonary disease. The most common baseline symptom was cough (95%), followed by fatigue (87%), weight loss (86%), loss of appetite (78%), fever (77%), chest pain (75%), night sweats (75%), and dyspnea (67%). The most common location of care-seeking prior to diagnosis was at a pharmacy (33%), followed by private practice (29%), private hospital (24%), and public district/provincial hospital (23%). Four individuals sought care from a traditional healer. Among PWH, the most common care-seeking location was private practice (31%), followed by a pharmacy (25%). Among participants without HIV, 36% sought care at a pharmacy.

**Table 1.** Sociodemographic and Clinical Characteristics of Participants

Characteristic	All (n = 264)	PWH (n = 81)	HIV Negative (n = 183)
Age, y, median (IQR)	33 (25–43)	40 (34–49)	30 (23–39)
Sex			
Male	176 (66.7)	43 (53.1)	133 (72.7)
Female	88 (33.3)	38 (46.9)	50 (27.3)
Study site			
Eldoret, Kenya	171 (64.8)	41 (50.6)	130 (71.0)
Mbarara, Uganda	93 (35.2)	40 (49.4)	53 (29.0)
On ART at TB diagnosis^a^	…	54 (66.7)	NA
CD4 count at TB diagnosis, cells/μL, median (IQR)^a^	…	121 (45–293)	NA
HIV viral load			
Suppressed (<75 copies/mL)^a^	…	15 (18.5)	NA
Not suppressed (≥75 copies/mL)^a^	…	17 (21.0)	NA
No HIV viral load^a^	…	49 (60.5)	NA
Place of residence			
Urban	134 (51.0)	43 (53.1)	91 (49.7)
Rural	130 (49.0)	38 (46.9)	92 (50.3)
Highest educational level			
None	11 (4.2)	9 (11.1)	2 (1.1)
Primary	86 (32.6)	33 (40.7)	53 (29.0)
Secondary	109 (41.2)	28 (34.6)	81 (44.3)
Postsecondary	58 (22.0)	11 (13.6)	47 (25.6)
Marital status			
Single	112 (42.4)	18 (22.2)	94 (51.4)
Married	93 (35.2)	26 (32.1)	67 (36.6)
Widowed	11 (4.2)	8 (9.9)	3 (1.6)
Divorced	42 (15.9)	2 (2.5)	4 (2.2)
Separated	6 (2.3)	27 (33.3)	15 (8.2)
Previous TB disease	57 (22.0)	25 (30.9)	32 (17)
Outcome of previous TB			
Successful treatment^b^	48 (84.2)	21 (84)	27 (84.4)
Poor outcome^c^	9 (15.8)	4 (16)	5 (15.6)
Known contact with TB	55 (20.8)	11 (13.6)	44 (24.0)
Type of contact with TB			
Household	35 (63.6)	8 (72.7)	27 (61.3)
Nonhousehold	19 (34.6)	3 (27.3)	16 (36.4)
Missing	1 (1.8)	0 (0.0)	1 (2.3)
Location(s) of care-seeking prior to diagnosis^d^			
Primary clinic	57 (21.6)	13 (16.1)	44 (24.0)
Public district/provincial hospital	61 (23.1)	11 (13.6)	50 (27.3)
Public teaching/referral hospital	50 (18.9)	15 (18.5)	35 (19.1)
Private practice	76 (28.8)	25 (30.9)	51 (27.9)
Private hospital	63 (23.9)	11 (13.6)	52 (28.4)
Pharmacy/dispensary	86 (32.6)	20 (24.7)	66 (36.1)
Self-management	24 (9.1)	5 (6.2)	19 (10.4)
Traditional healer	4 (1.5)	1 (1.2)	3 (1.6)
Other	5 (1.9)	1 (1.2)	4 (2.2)
TB diagnosis type			
Pulmonary alone	261 (98.9)	80 (98.8)	181 (98.9)
Pulmonary and extrapulmonary	3 (1.1)	1 (1.2)	2 (1.1)
Microbiologic confirmation			
Clinical diagnosis	29 (11.0)	11 (13.6)	16 (8.7)
Microbiologically confirmed^e^	235 (89.0)	70 (86.4)	165 (90.2)
Baseline TB symptoms^f^	n = 263	n = 80	n = 183
Fever	203 (77.2)	64 (80.0)	139 (76.0)
Cough	251 (95.4)	76 (95.0)	175 (95.6)
Weight loss	225 (85.6)	71 (88.8)	154 (84.2)
Night sweats	197 (74.9)	63 (78.8)	134 (73.2)
Chest pain	197 (74.9)	59 (73.8)	138 (75.4)
Dyspnea	176 (67.2)	55 (69.6)	121 (66.1)
Abdominal pain	83 (31.6)	28 (35.0)	55 (30.1)
Fatigue	228 (86.7)	75 (93.8)	153 (83.6)
Loss of appetite	204 (77.6)	66 (82.5)	138 (75.4)
Hospitalized at TB presentation	28 (10.6)	14 (17.3)	14 (7.7)
Food insecurity (HFIAS)			
None	81 (30.7)	21 (25.9)	60 (32.8)
Mild	51 (19.3)	17 (21.0)	34 (18.6)
Moderate	87 (32.9)	24 (29.6)	63 (34.4)
Severe	44 (16.7)	19 (23.5)	25 (13.7)
Missing	1 (0.4)	0 (0.0)	1 (0.6)
TB stigma standardized score, median (IQR)^g^	1.7 (1.3–2.2)	1.8 (1.3–2.3)	1.7 (1.3–2.1)
Perceived community HIV stigma standardized score, median (IQR)^g^	2.1 (1.4–2.7)	2.1 (1.5–2.7)	2.0 (1.4–2.7)
Felt/experienced HIV stigma standardized score, median (IQR)^a,g^	…	3.1 (2.7–3.4)	NA

Data are presented as No. (%) unless otherwise indicated.

Abbreviations: ART, antiretroviral therapy; HFIAS, Household Food Insecurity Access Scale; HIV, human immunodeficiency virus; IQR, interquartile range; NA, not applicable; PWH, people with human immunodeficiency virus; TB, tuberculosis.

^a^For PWH only.

^b^Previous successful TB treatment outcome is defined as treatment completion or cure.

^c^Previous poor TB treatment outcome is defined as loss to follow-up or treatment failure.

^d^Participants can select multiple locations of care-seeking.

^e^Microbiologic confirmation was by sputum Xpert MTB/RIF (n = 189), sputum acid-fast bacilli smear (n = 38), and/or urine lipoarabinomannan (LAM) test (n = 34, used in PWH).

^f^Symptoms were missing for 1 participant.

^g^Stigma scores ranged from 0 to 4 (0 = strong disagreement, 2 = no opinion, 4 = strong agreement with stigma statements), with higher scores representing greater stigma. Scores were standardized by taking the mean score of all items within each domain.

### Timing of Initial Presentation and TB Treatment Initiation

**Figure 2.**
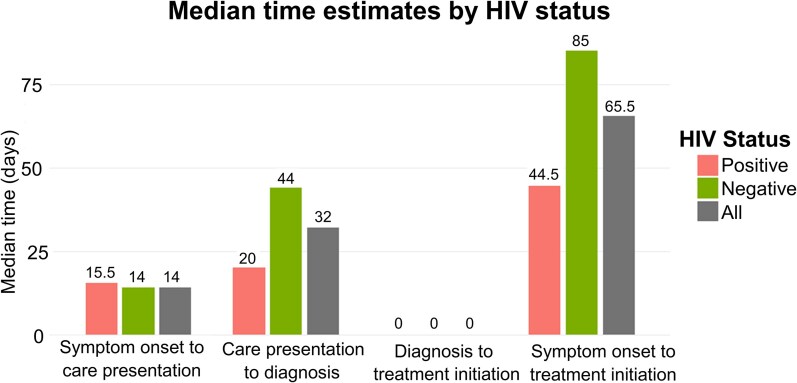
Median durations for key events in the tuberculosis (TB) diagnosis and treatment initiation cascade, by human immunodeficiency virus (HIV) status. Median durations are presented (in days) for key events in the TB diagnosis and treatment initiation cascade described in this study: from symptom onset to first care presentation; from first care presentation to TB diagnosis; from TB diagnosis to treatment initiation; and overall duration from TB symptom onset to treatment initiation. Median durations are presented for people with HIV, individuals without HIV, and all participants.

**Figure 3.**
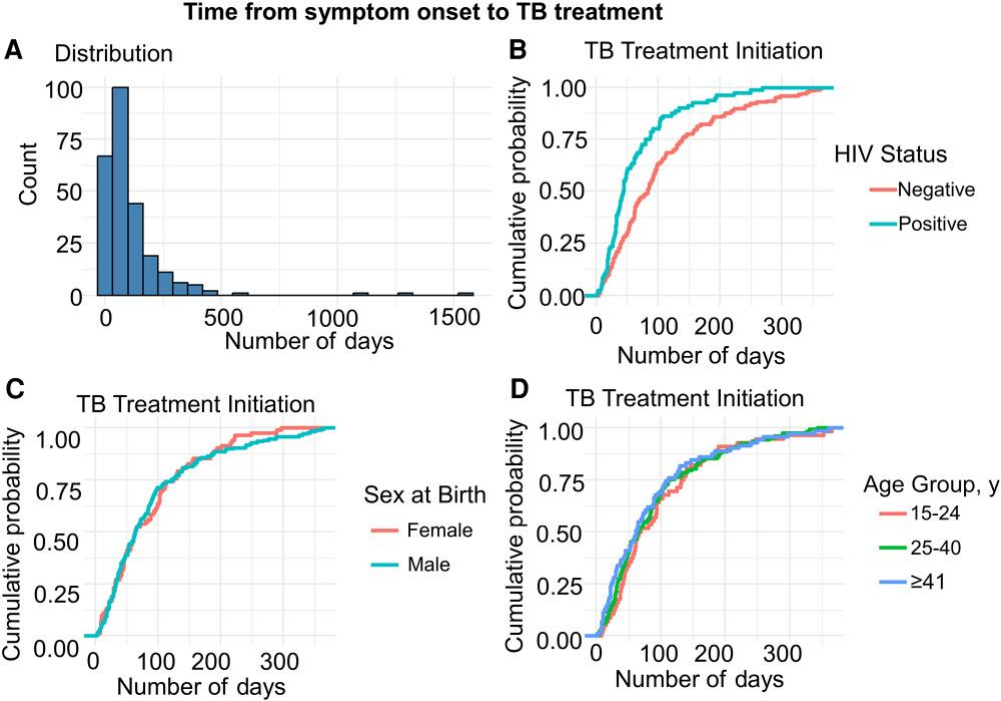
Distribution of time (in days) from tuberculosis (TB) symptom onset to treatment initiation, along with empirical cumulative distribution functions (ECDFs) stratified by key characteristics. *A*, Distribution of days from symptom onset to TB treatment initiation for all participants. ECDFs are presented to visualize timing from symptom onset to TB treatment initiation, stratified by human immunodeficiency virus (HIV) status (*B*), sex (*C*), and age group (*D*).

**Figure 4.**
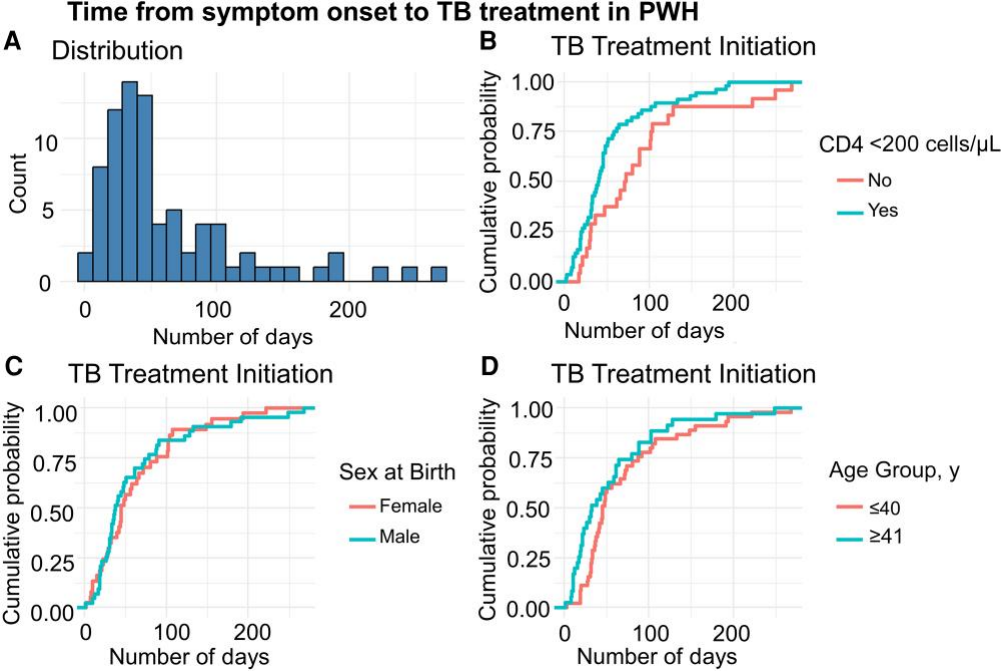
Distribution of time (in days) from tuberculosis (TB) symptom onset to treatment initiation among people with human immunodeficiency virus (PWH), along with empirical cumulative distribution functions (ECDFs) stratified by key characteristics. Among PWH only, distribution of days from symptom onset to TB treatment initiation for all PWH (*A*). ECDFs are presented to visualize timing from symptom onset to TB treatment initiation, stratified by CD4 (*B*), sex (*C*), and age group (*D*), among PWH.

The median overall duration from symptom onset to TB treatment initiation was 65.5 days (IQR, 33–131 days) (Figures 2 and 3). The median duration from symptom onset to initial care presentation was 14 days (IQR, 4.0–44.0 days) and from initial presentation to TB diagnosis was 32.0 days (IQR, 8–78 days). Once a TB diagnosis was made, the median duration from TB diagnosis to treatment initiation was 0 days (IQR, 0.0–0.0 days). The median number of healthcare visits from symptom onset to TB diagnosis was 2 (IQR, 1–3 days). For PWH, the median overall duration from symptom onset to TB treatment initiation was 44.5 days (IQR, 26.5–83.5 days) (Figure 4); from symptom onset to initial care presentation was 15.5 days (IQR, 3–33 days); from initial presentation to TB diagnosis was 20 days (IQR, 3.0–40.0 days); and from TB diagnosis to treatment initiation was 0 days (IQR, 0.0–1.0 days). Among people with negative HIV status, median overall duration from symptom onset to TB treatment initiation was 85 days (IQR, 42–155 days); from symptom onset to initial care presentation was 14 days (IQR, 5–50 days); from initial presentation to TB diagnosis was 43 days (IQR, 8.0–95.0 days); and from TB diagnosis to treatment initiation was 0 days (IQR, 0.0–0.0 days).

### Associations With Delay to Treatment Initiation

Longer overall duration from TB symptom onset to TB treatment initiation was associated with female sex (adjusted IRR [aIRR], 1.27 [95% confidence interval {CI}, 1.00–1.61]), previous care-seeking at a pharmacy (aIRR, 1.53 [95% CI, 1.22–1.92]), and presence of dyspnea (aIRR, 1.43 [95% CI, 1.09–1.86]) or fatigue (aIRR, 1.63 [95% CI, 1.11–2.37]) (Table 2). Shorter duration from TB symptom onset to treatment initiation was associated with HIV-positive status (aIRR, 0.50 [95% CI, .39–.64]). Among PWH, shorter duration from TB symptom onset to treatment initiation was associated with CD4 count <200 cells/μL (aIRR, 0.63 [95% CI, .43–.93]) (Table 3).

**Table 2.** Robust Binomial Regression of Time From Tuberculosis Symptom Onset to Treatment Initiation for All Participants (N = 258)

Characteristic	Univariable			Multivariable		
	IRR	(95% CI)	*P* Value	IRR	(95% CI)	*P* Value
Age, y			.83			.67
15–24 (Reference)	…	…		…	…	
25–40	1.03	(.76–1.39)		1.13	(.85–1.50)	
≥41	0.95	(.67–1.33)		1.14	(.82–1.57)	
Female sex	1.00	(.78–1.29)	.99	1.27	(1.00–1.61)	.**049**
Urban residence	0.89	(.70–1.13)	.35	0.88	(.70–1.10)	.25
Eldoret, Kenya study site	1.18	(.91–1.51)	.20	1.22	(.93–1.59)	.15
Hospitalized at diagnosis	1.09	(.76–1.63)	.65	1.06	(.75–1.52)	.76
Living with HIV	0.46	(.36–.60)	**<**.**001**	0.50	(.39–.64)	**<**.**001**
Microbiologically confirmed	1.05	(.70–1.53)	.80	1.14	(.78–1.63)	.48
Food insecurity (HFIAS)			.16			.26
None (Reference)	…	…		…	…	
Mild	0.91	(.65–1.29)		1.11	(.81–1.53)	
Moderate	0.92	(.68–1.24)		0.94	(.70–1.25)	
Severe	0.65	(.45–.95)		0.77	(.54–1.09)	
Previously treated for TB	0.95	(.71–1.30)	.75	0.96	(.72–1.29)	.79
Known TB contact	1.05	(.78–1.43)	.77	0.93	(.71–1.23)	.61
TB stigma	0.91	(.75–1.11)	.34	0.95	(.77–1.17)	.62
Perceived community HIV stigma	0.98	(.86–1.13)	.80	1.02	(.89–1.17)	.80
Dyspnea at diagnosis	1.46	(1.13–1.88)	.**005**	1.43	(1.09–1.86)	.**009**
Fatigue at diagnosis	1.57	(1.09–2.21)	.**017**	1.63	(1.11–2.37)	.**014**
Additional symptoms at baseline	1.12	(1.04–1.21)	.**005**	1.06	(.89–1.17)	.21
Care-seeking with traditional healer	1.58	(.68–4.98)	.32	2.09	(.95–5.49)	.067
Care-seeking at a pharmacy	1.81	(1.42–2.32)	**<**.**001**	1.53	(1.22–1.92)	**<**.**001**

Bolded values indicate statistical significance (*P* < .05).

Abbreviations: CI, confidence interval; HFIAS, Household Food Insecurity Access Scale; HIV, human immunodeficiency virus; IRR, incidence rate ratio; TB, tuberculosis.

**Table 3.** Robust Binomial Regression Analysis of Time From Tuberculosis Symptom Onset to Treatment Initiation for People With HIV Only (n = 80)

Characteristic	Univariable			Multivariable		
	IRR	(95% CI)	*P* Value	IRR	(95% CI)	*P* Value
Age ≥41	0.76	(.53–1.11)	.15	0.70	(.49–1.00)	.051
Female sex	1.00	(.69–1.44)	.98	1.01	(.71–1.45)	.94
Urban residence	0.95	(.66–1.37)	.79	0.99	(.70–1.40)	.94
Fatigue at diagnosis	2.56	(1.11–5.09)	.**030**	1.62	(.75–3.22)	.21
Dyspnea at diagnosis	1.45	(.97–2.12)	.070	1.37	(.91–2.02)	.13
Perceived community HIV stigma	1.22	(.98–1.51)	.076	1.16	(.89–1.51)	.28
Felt HIV stigma	0.98	(.73–1.28)	.90	0.81	(.56–1.12)	.21
CD4 count <200 cells/μL	0.60	(.41–.88)	.**008**	0.63	(.43–.93)	.**021**

Bolded values indicate statistical significance (*P* < .05).

Abbreviations: CI, confidence interval; HIV, human immunodeficiency virus; IRR, incidence rate ratio.

## DISCUSSION

In this prospective cohort study of individuals with pulmonary TB in Eldoret, Kenya, and Mbarara, Uganda, we found generally prolonged durations from recalled TB symptom onset to TB treatment initiation, with an overall median of 66 days before TB was ultimately diagnosed and treated—with significantly longer durations demonstrated for certain groups. Delays to TB treatment, particularly among HIV-negative and female individuals, point to the need for increased ascertainment of TB at lower levels of care and community settings. Given that delayed TB diagnosis contributes to poor clinical outcomes [10–12] and TB transmission [8, 9], this remains a critical target to make progress in TB elimination. Complementary tailored strategies are needed to ensure access for those most at risk for TB care delays.

Despite longstanding awareness and efforts to address gaps in TB detection and treatment, including scale-up of Xpert MTB/RIF for TB diagnosis since 2010, delays to TB care in this cohort were broadly similar to those described in past studies. A 2008 systematic review found that people with TB experienced a total diagnostic delay of 60–90 days in most countries, including low or high TB-endemic settings, with variation in whether time to first healthcare visit or time from first healthcare visit to diagnosis was the greater contributor to delay [4]. A 2017 systematic review of pulmonary TB diagnosis in low- and middle-income countries reported a median total delay of 67 days [3]. While contributions to delay may vary across time and setting, delayed TB diagnosis and treatment initiation is unfortunately common and enduring.

In this cohort, PWH experienced shorter time from symptom onset to TB treatment initiation. Time to initial presentation to care was similar between PWH and those who were HIV negative, but PWH had substantially shorter time from care presentation to diagnosis. PWH were less likely to have sought care at a pharmacy than people who were HIV negative. In previous studies, living with HIV has been associated with both longer [25, 26] and shorter [27, 28] time to TB diagnosis and treatment initiation, owing to variations in TB-HIV care access and integration across study time periods and contexts [26, 27]. For participants in our study, integrated TB screening/diagnosis at HIV care programs at these sites likely facilitated more timely recognition of TB and initiation of treatment [29].

Delayed TB care in this study was associated with initial care-seeking at a pharmacy. Previous studies have consistently found TB care delays to be associated with care-seeking at pharmacies [30, 31] and also traditional healers [3, 4]. Increasing access to TB diagnosis in community and primary healthcare settings, including dedicated pharmacy interventions, may facilitate timely care. A recent study in India found that TB screening and referral within pharmacies was feasible and highly impactful for TB notifications [32].

Longer duration from symptom onset was associated with dyspnea and fatigue by time of assessment at TB diagnosis, likely representing more profound illness after delays to presentation. Previous systematic reviews found that nonspecific symptoms such as cough, fever, and night sweats were often attributed to other respiratory infections, whereas hemoptysis prompted TB testing and care [3, 5]. Our findings highlight the need for more timely TB diagnosis prior to development of more severe symptoms. In contrast, among PWH, those with CD4 count <200 cells/μL had shorter time to treatment initiation than PWH with higher CD4 counts. This finding likely relates to more prompt TB evaluations and treatment initiation among individuals with advanced HIV.

Female sex was associated with delay to TB treatment initiation. Previous studies have been mixed—finding delays associated with male sex [33–35] or female sex [36–38]—with variation in study settings, populations, and measures for TB care entry. A mixed-methods systematic review and meta-analysis found that female sex was associated with delays in care-seeking, with disproportionate impacts of unemployment, low TB knowledge, and transportation barriers to care [5]. In qualitative studies, women reported economic constraints and power imbalances as barriers to care-seeking [5]. A systematic review of qualitative studies found that barriers affected men and women differently, with women experiencing financial and physical dependence, lower literacy, and household stigma, while men faced work-related financial and physical barriers and community-based stigma [39]. While men are disproportionately affected by TB generally, among PWH in Africa, age-standardized TB incidence rates are greater among women [40]. Despite such differences in TB incidence and care access, TB programs, policies, and guidelines may not take sex into account [41]. Our study highlights the need for tailored strategies for TB case-finding and further research into TB care disparities by sex.

We did not find an association between TB or HIV stigma and delayed TB care. Stigma is detrimental to TB treatment adherence [42–44] and well-being [43, 45]. It is perceived as a barrier to TB diagnosis, but research has been mixed [46]. TB stigma scale scores in this cohort were relatively low, with most participants disagreeing with stigma statements and not reporting stigma as a deterrent to care-seeking. Limitations to existing TB stigma measures may also impact these findings. Felt HIV stigma was high among PWH, but not associated with delayed TB care. HIV stigma profoundly influences HIV care engagement [47] and well-being [48], but it may have less direct impacts on initiation of TB treatment. Access to TB diagnosis may be considered a central driver of delayed TB care.

In early 2026, the WHO issued new recommendations for near-point-of-care molecular tests for TB, tongue-swab sampling, and sputum pooling strategies—all designed to decentralize TB diagnosis in community settings [49]. Our findings point to the necessity for such innovations, given persistent delays in care and gaps in diagnostic access. Implementing these strategies in TB-endemic settings may be critical to ensuring timely TB care. Yet precisely as these innovations offer new promise for expanding TB diagnosis and accelerating elimination efforts, a global health funding crisis threatens to widen the gaps in TB care access identified in this study [50]. Maintaining momentum behind proven and emerging strategies for TB case-finding has never been more critical.

There are multiple limitations to this study. Self-reported data on symptom onset and care-seeking is subject to recall bias. Further, individuals with subclinical TB may have infectious TB prior to awareness of symptoms [51]. Therefore, the care delays noted in our study may underrepresent the scope of the problem, with individuals potentially being infectious for a longer period prior to TB treatment initiation. The modest sample size of PWH limited analysis within this subpopulation to exploratory analysis with a limited number of variables. Because our study took place at major referral hospitals, findings may not be generalizable to facilities at other care levels. However, a strength of the study is that its performance at referral hospitals in 2 countries, each with large catchment areas with diverse populations, ensures that a full range of participant characteristics and care trajectories are included, supporting robust analyses and informing care in these settings. Our approach is otherwise strengthened by a prospective design, with systematic ascertainment of symptom duration at entry into TB care, along with performance of several standardized questionnaires including for sociodemographic and behavioral factors related to social determinants of health.

In conclusion, we identified substantial delays from TB symptom onset to treatment initiation in this East African cohort of individuals with pulmonary TB. Disproportionately longer delays experienced by women, HIV-negative individuals, and individuals seeking care at pharmacies highlight the urgent need to increase access to TB diagnosis at the community level, including through tailored approaches. Increasing TB awareness and expanding access to diagnostics—including through scale-up of next-generation near-point-of-care molecular tests and decentralized testing strategies—are essential steps toward TB elimination.
